# Prediction of aortic dilatation in surgically repaired type A dissection: A longitudinal study using computational fluid dynamics

**DOI:** 10.1016/j.xjon.2022.01.019

**Published:** 2022-02-09

**Authors:** Yu Zhu, Xiao Yun Xu, Ulrich Rosendahl, John Pepper, Saeed Mirsadraee

**Affiliations:** a Department of Chemical Engineering, Imperial College London, London, United Kingdom; b National Heart and Lung Institute, Imperial College London, London, United Kingdom; c Department of Cardiac Surgery, Royal Brompton and Harefield Hospitals, London, United Kingdom; d Department of Radiology, Royal Brompton and Harefield Hospitals, London, United Kingdom

**Keywords:** type A aortic dissection, aortic dilatation, computational fluid dynamics, luminal pressure difference, CFD, computational fluid dynamics, CT, computed tomography, CTA, computed tomography angiography, FL, false lumen, ROC, receiver operating characteristic, TAAD, type A aortic dissection, TBAD, type B aortic dissection, TL, true lumen

## Abstract

**Objective:**

To examine the role of a key hemodynamic parameter, namely the true and false lumen pressure difference, to predict progressive aortic dilatation following type A aortic dissection (TAAD) repair.

**Methods:**

Four patients with surgically repaired TAAD with multiple follow-up computed tomography angiography scans (4-5 scans per patient; N = 18) were included. Through-plane diameter of the residual native thoracic aorta was measured in various aortic segments during the follow up period (mean follow-up: 49.6 ± 31.2 months). Computational flow analysis was performed to estimate true and false lumen pressure difference at the same locations and the correlation with aortic size change was studied using a linear mixed effects model.

**Results:**

Greater pressure difference between the true and false lumen was consistent with greater aortic diameter expansion during the follow up period (linear mixed effects analysis; coefficient, 0.26; 95% confidence interval, 0.15-0.37; *P* < .001). Based on our limited data points, a pressure difference higher than 5 mm Hg might cause unstable aortic growth.

**Conclusions:**

Computational fluid dynamic assessment of standard aortic computed tomography angiography offers a noninvasive technique that predicts the risk of aortic dilatation following TAAD. The technique may be used to plan closer observation or intervention in high-risk patients.

## Graphical abstract

**Figure:**
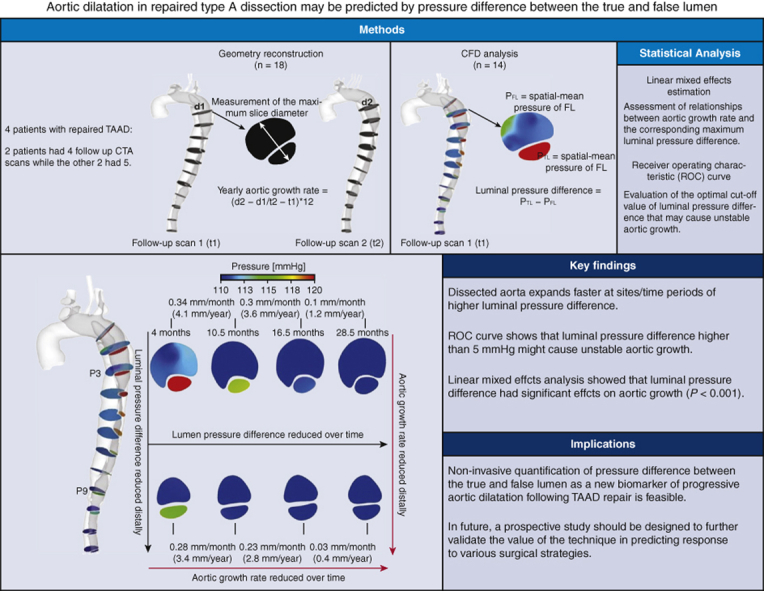

**Figure:**
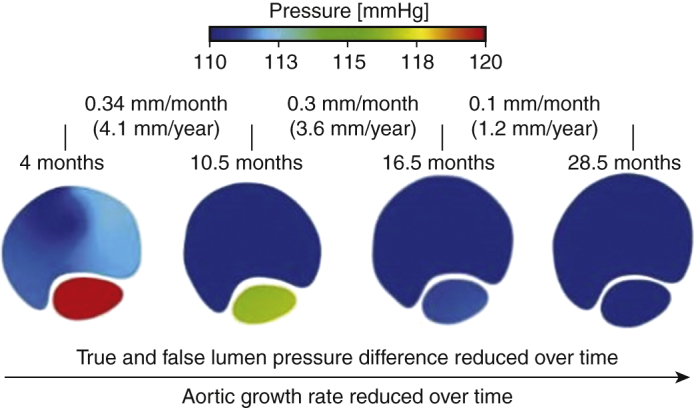
Dissected aorta expands faster at time periods of greater luminal pressure difference.

**Central Message**

Measuring the pressure difference between the true and false lumens using a CTA-derived flow modeling technique predicts the risk of aortic dilatation over time in repaired type A aortic dissection.

**Perspective**

Noninvasive quantification of pressure difference between the true and false lumen as a new biomarker of progressive aortic dilatation following type A aortic dissection repair is feasible. This technique offers a new approach in the follow-up and risk stratification of type A aortic dissection.

Stanford type A aortic dissection (TAAD) is treated with a variety of surgical and endovascular repair techniques, from just replacing the ascending aorta to a more complex total arch replacement. Although isolated replacement of the ascending aorta has the lowest reported immediate perioperative risk and mortality,^1^ the reported prevalence of medium and late complications such as aneurysmal dilatation of the remaining dissected aorta is greater.^2^, ^3^, ^4^, ^5^, ^6^ The rupture of the residual dissected aorta is one of the most common causes of late death, with a reported mortality rate of 29.3%.^7^

Anatomical features, such as greater aortic diameter,^2^^,^^3^ false lumen patency,^2^, ^3^, ^4^ and larger number of proximal communications (tears) in the arch^5^ were associated with aortic growth, but these studies could not explain why aortic dilatation occurs nor could identify variables that reliably predict the risk of future aortic dilatation.

Hemodynamic parameters, such as total intraluminal pressure and wall shear stress, have been reported to directly affect the function of the aortic wall,^8^^,^^9^ which may play a role in the progression of aneurysmal dilatation. Computational fluid dynamics (CFD) based on clinical imaging studies has become an indispensable tool for studies of blood flow in the cardiovascular system^10^ since it can provide a sufficiently accurate prediction of pressure and shear stress that are difficult to measure in vivo.^11^^,^^12^ By incorporating patient-specific boundary conditions in their CFD analysis of a type B aortic dissection (TBAD), Pirola and colleagues^12^ showed an overall good agreement between predicted flow and pressure and in vivo measurements.

Recent studies reported that greater luminal pressure difference between the true lumen (TL) and false lumen (FL)^13^ and peak systolic, mid- and late systolic velocity magnitudes in the tears (centerline of each tear)^14^ were associated with progressive aortic dilatation following TAAD repair. However, in both studies, CFD simulations were only performed for geometries reconstructed from the baseline computed tomography angiography (CTA) images, whereas variable aortic changes over follow-up scans were not taken into consideration.

In this study, we examined patients with repaired TAAD and multiple follow-up CTA images to see whether CFD estimation of luminal pressures would reliably predict the risk of aortic dilatation over time.

## Methods

### Study Design

This study was approved by the institutional committee of Health Research Authority (HRA) and Health and Care Research Wales (HCRW) on May 4, 2020 (ref: 20/WM/0145), and the need for informed patients' consent was waived.

**Figure 1:**
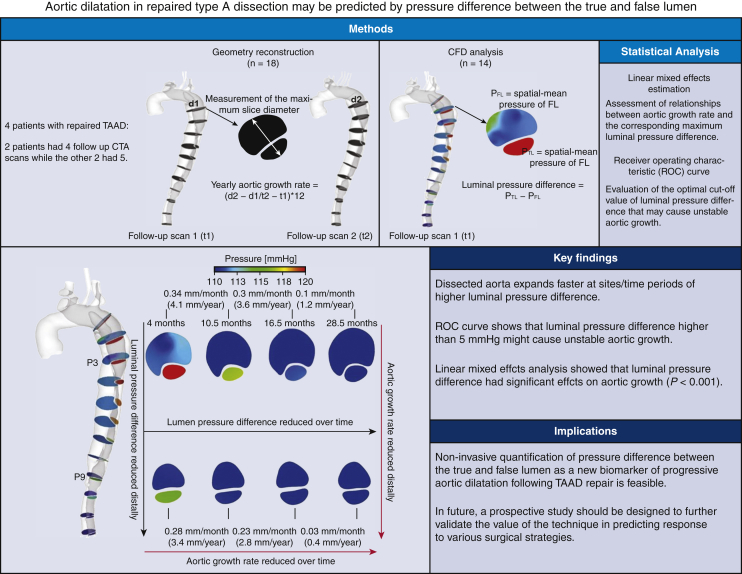
Four patients with surgically repaired type A aortic dissection (*TAAD*) with multiple follow-up computed tomography angiography (*CTA*) images were included in this study. Despite the small number of patients, 18 dissection geometries were reconstructed by making use of all follow-up CTA images, after which the yearly aortic growth rate (mm/year) was evaluated among each cross-sectional plane at every follow-up scan. Computational flow analysis was performed for 14 of 18 geometries, because geometries reconstructed from the last CTA scans were served as comparative purpose only. A key hemodynamic factor, namely, the pressure difference between the true and false lumen, was evaluated at the same cross-sectional planes. The relationship between aortic growth rate and the corresponding luminal pressure difference was then assessed using a linear mixed effects model. Results of the study revealed that luminal pressure difference had a significant effect on aortic growth, suggesting its role as a predictor of aortic dilatation in patients with repaired TAAD. Moreover, a receiver operating characteristic curve shows that a maximum pressure difference of 5 mm Hg (or greater) might cause unstable growth (yearly aortic growth rate >2.9 mm). *CFD*, Computational fluid dynamic; *TL*, true lumen; *FL*, false lumen.

This retrospective study was based on a validated database of patients with repaired TAAD at the Royal Brompton and Harefield Hospitals. We identified 4 patients who had undergone multiple follow-up CTA scans with sufficient image quality for geometry reconstruction. Of these 4, 2 patients had 4 follow-up CTA studies, and the other 2 had 5. A total number of 18 computed tomography (CT) scans were used for patient-specific geometry reconstruction. The last scan from each patient was used for comparative purpose only and thus the computational flow analysis was performed for 14 of 18 generated geometries. For each patient, aortic size change in various aortic segments was evaluated between each 2 consecutive follow-up CTA scans. A key hemodynamic parameter, pressure difference between the TL and FL (PD_TL-FL_) was calculated for the same locations and the correlation with aortic size change was analyzed. An overview of the study is presented in Figure 1 as well as Video 1.

### Geometry Reconstruction and Morphologic Measurements

**Figure E1:**
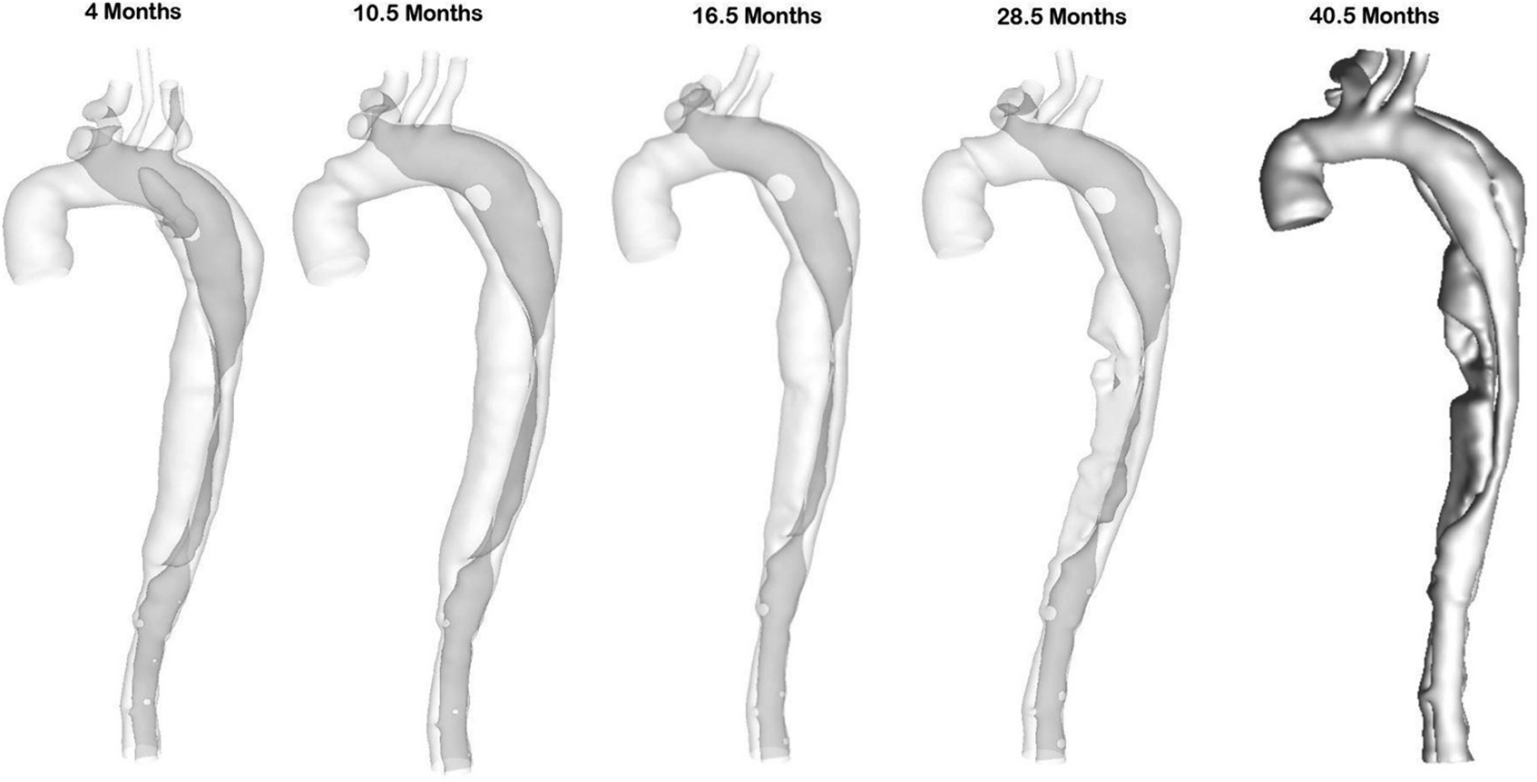
Geometry reconstructions for patient 1. All reconstructed geometries, except the last one, were used for computational flow analysis and thus were displayed in a transparent view to show the tears.

**Figure E2:**
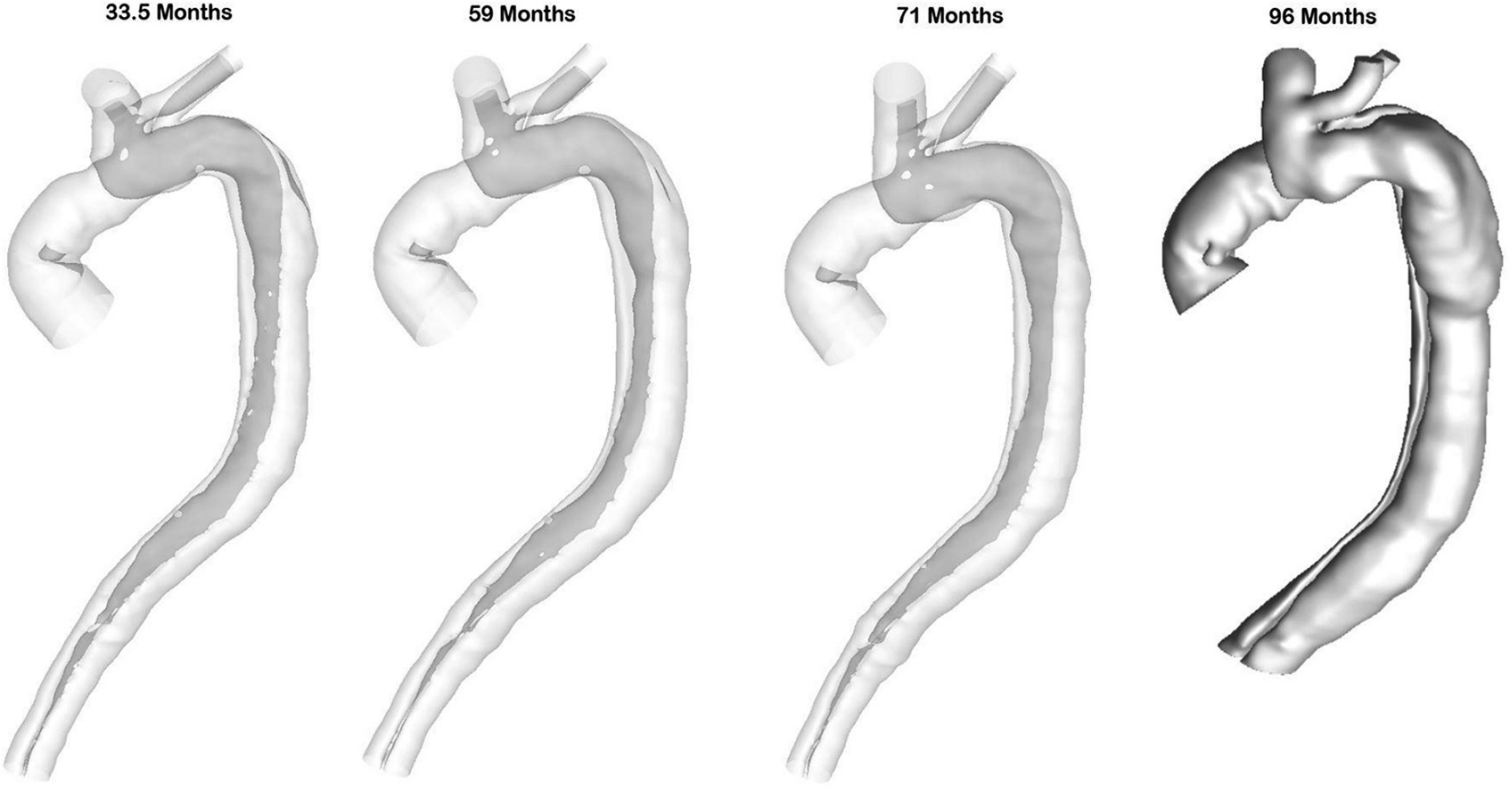
Geometry reconstructions for patient 2. All reconstructed geometries, except the last one, were used for computational flow analysis and thus were displayed in a transparent view to show the tears. It should be noted that the last reconstructed geometry includes only the thoracic aorta due to inadequate computed tomography angiography image coverage.

**Figure E3:**
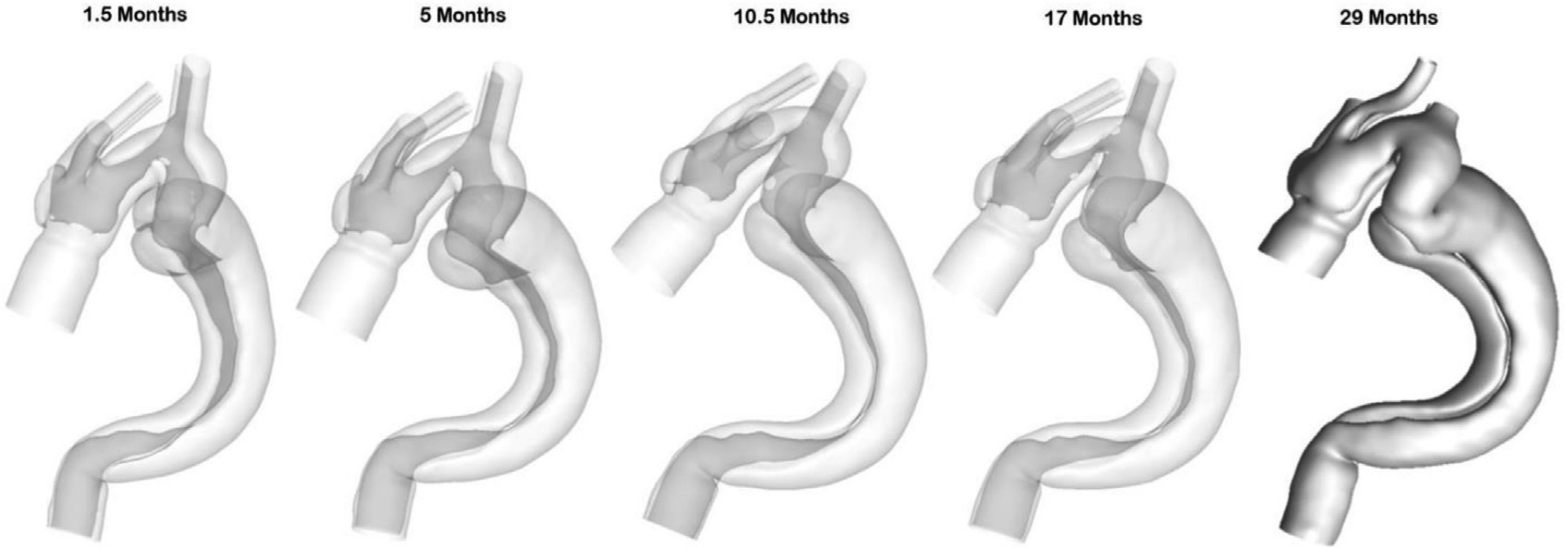
Geometry reconstructions for patient 3. All reconstructed geometries, except the last one, were used for computational flow analysis and thus were displayed in a transparent view to show the tears.

**Figure E4:**
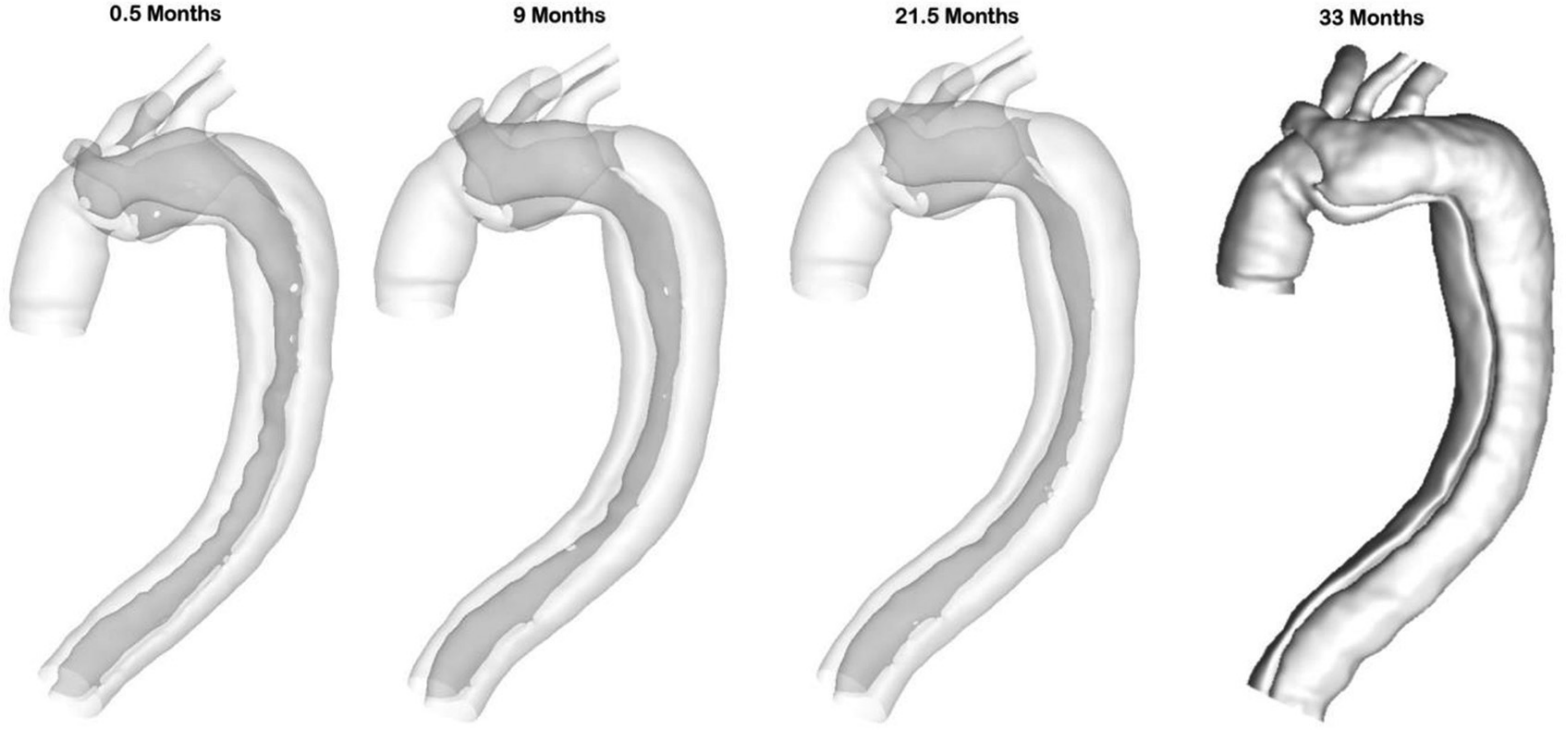
Geometry reconstructions for patient 4. All reconstructed geometries, except the last one, were used for computational flow analysis and thus were displayed in a transparent view to show the tears.

CTA scans in 3 patients were performed on a 128-detector row scanner (Siemens Medical Solutions), and the images were reconstructed with 0.75-mm slice thickness and 0.5-mm slice increment. The other 1 patient was examined by a 64-detector row scanner (GE Healthcare), where the slice thickness and increment of CTA images were 0.625 mm. Patient-specific geometries were reconstructed from the CTA data using an image analysis software Mimics 22.0 (Materialise HQ). All reconstructed geometries can be found in Appendix 1 (Figure E1, Figure E2, Figure E3, Figure E4).

To ensure reconstruction accuracy, for each geometry, cross-sectional contours were extracted from the reconstructed 3-dimensional surface, which were then mapped back to the raw CT images to check if the contours well presented the edges of the aortic lumen. All reconstructions were performed by a well-trained operator to minimize human errors, and reconstructed geometries were reviewed by a radiologist with over 10-year experience in cardiovascular imaging. Moreover, an intraoperator reproducibility study was carried out, demonstrating a reproducibility of greater than 96% in all key geometric measures with 98.4% in aortic diameter measurement. The detailed results are summarized in Table E1 (Appendix 2).

For patients 1 and 2, both the thoracic and abdominal aorta were included in the reconstruction, whereas only the thoracic aorta was involved for patients 3 and 4 due to inadequate CTA image coverage.

**Figure 2:**
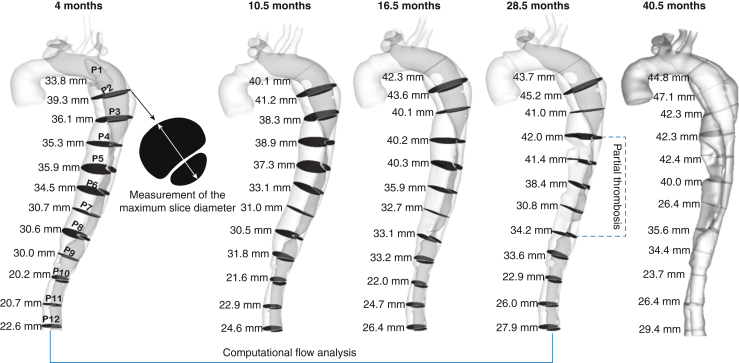
Illustration of reconstructed geometries for patient 1 based on all follow-up computed tomography (CT) data. For each geometry, 12 cross-sectional planes perpendicular to the centerline were created, after which the maximum short-axis diameters were measured. Computational fluid dynamic (CFD) simulations were performed for all patient-specific models except for the last one, which was served for comparative purpose only. Moreover, the partial thrombosed region of FL (eg, P4-P8) was not reconstructed for CFD simulations, since there is no blood flow, but it was taken into consideration when we measured aortic diameters.

A centerline was fitted for each reconstructed geometry using Mimics, after which 8 or 12 cross-sectional planes perpendicular to the centerline were created, depending on the aortic length. The first plane (P1) was selected at 2 cm distal to the origin of left subclavian artery and P2 to P8/12 were evenly spaced below P1 with an interval of 3 cm. As shown in Figure 2, the maximum diameter was taken at each plane and compared between various time periods. Monthly aortic growth rate at each cross-sectional plane was first calculated by dividing the change in diameter by the time interval between 2 consecutive follow-up CTA scans. The equivalent yearly growth rate was then evaluated by multiplying the monthly aortic growth rate by 12. The aortic segments grew >2.9 mm/year were classified as unstable growth.^15^

Other key geometrical parameters, such as primary entry tear size, number of re-entry tears, and TL and FL volumes, were also measured and summarized in Table 1. It should be noted that the “primary entry tear” referred to in this paper is the most proximal tear in the residual dissected aorta whereas the initial primary tear in the ascending aorta was resected during surgery. The re-entry tears were identified on the CTA images as communications between the TL and FL, located distally to the primary entry tear.

**Table 1:** Measurements of key geometric parameters for each patient at various scans

Geometric parameters	Patient 1				Patient 2			Patient 3				Patient 4		
	Follow-up scan time to surgery, mo													
	4	10.5	16.5	28.5	33.5	59	71	1.5	5	10.5	17	0.5	9	21.5
Primary tear size														
Area, mm^2^	110	110	150	170	24	19	19	42	69	71	71	22	15	13
Number of re-entry tears	6	6	7	8	25	18	18	7	5	3	8	15	9	11
Volume ratio of FL/TL	3.3	3.1	3.1	3.1	3.2	3.9	4.3	2.2	2.2	2.2	2.2	1.1	1.8	1.8
% Thrombus/FL	0	3	7	22	24	32	37	0	0	0	0	0	0	0
Volume change between 2 consecutive scans	#1- #2	#2-#3	#3-#4	#4-#5	#1- #2	#2-#3	#3-#4	#1 - #2	#2-#3	#3-#4	#4-#5	#1- #2	#2-#3	#3-#4
TL, %	17	0.5	17	20	1	3.1	N/A	4	1	0.2	5	−18	1	3
FL, %	10	12	6	8	21	15	N/A	6	2	8	−0.5	25	−2	−1

*FL*, False lumen; *TL*, true lumen; *N/A*, not applicable.

### CFD Model

Details of the computational methods, including geometry reconstruction, mesh generation, and the applied boundary conditions can be found in our previous study.^13^ The transitional turbulent blood flow^16^ through these patient-specific models were simulated by solving the Navier–Stokes equations with a finite volume-based solver (CFX 15; Ansys). Blood was assumed to be incompressible and Newtonian with a constant density of 1060 kg/m^3^ and dynamic viscosity of 4 mPa·s.

The simulation results were calculated and analyzed using CEI Ensight 10 (CEI Inc). Spatial mean pressure over a cardiac cycle were evaluated separately for the TL and FL, at the same cross-sectional planes that were created for aortic diameter measurements (Figure 2). Based on these cross-sectional pressure waveforms, differences between TL and FL pressures (PD_TL-FL_ = P_TL_–P_FL_) were calculated, and within each cross-sectional plane, the maximum PD_TL-FL_ over a cardiac cycle was determined.

### Statistical Analysis

**Figure E5:**
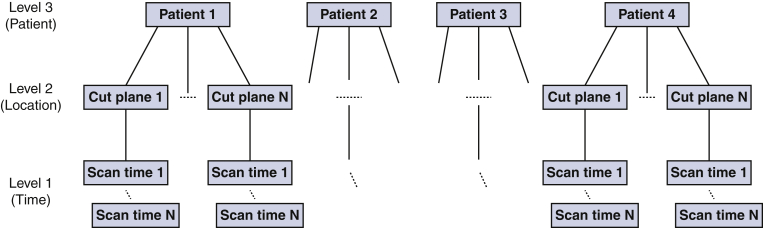
Illustration of 3-level data structure of linear mixed model. Follow-up time points of measurements (level 1 cluster) were nested within various locations (ie, cut planes) along the aorta (level 2 cluster), and then the locations were nested within each patient (level 3 cluster).

Aortic growth rate was evaluated and normalized as diameter change per year (Δ*D*/year) at each cross-sectional plane, after which a 3-level linear mixed effects model with restricted maximum likelihood estimation was performed to analyze the longitudinal data. A 3-level data structure (Figure E5) together with the corresponding equations can be found in Appendix 4. Follow-up time points and cut plane locations were treated as nominal data and defined as fixed effects. The main predictor, PD_TL-FL_, was also defined as a fixed effect, whereas the intercept was defined as the sole random effect with unstructured covariance being determined. The statistical analysis was carried out using SAS v. 9.4 (SAS Institute, Inc.). Moreover, a receiver operating characteristic (ROC) curve was fitted in SPSS v. 23.0 (IBM Corp.), in order to find the optimal cut-off value of PD_TL-FL_.

## Results

Table 2 shows the patient characteristics and the results of the image analysis are discussed case-by-case.

**Table 2:** A summary of patient characteristics

	Patient			
	1	2	3	4
Age, y	39	48	72	79
Sex	Male	Female	Female	Male
Comorbidities	Hypertension (controlled with antihypertensive medication)	Marfan syndrome, hypertension	Hypertension (controlled with antihypertensive medication), bicuspid aortic valve stenosis	Hypertension (controlled with antihypertensive medication)
Initial operation	Bentall procedure (mechanical valve)	Replacement of the aortic root and ascending aorta	Replacement of the aortic valve and ascending aorta	Replacement of the aortic root and ascending aorta
Surgery to first available follow-up CTA scan, mo	4	33.5	1.5	0.5
No. of postsurgery CTA scans	5	4	5	4

*CTA*, Computed tomography angiography.

### Patient 1

**Figure 3:**
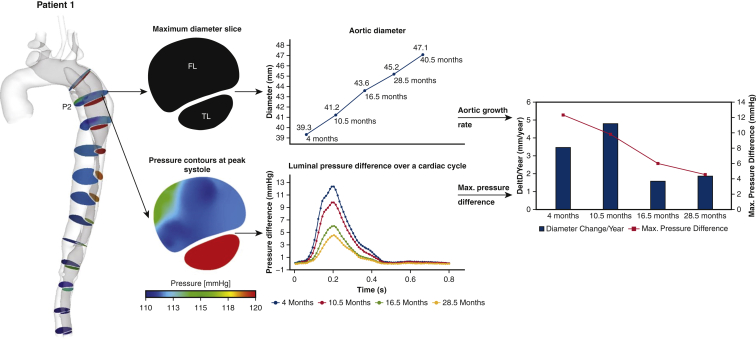
Illustration of aortic diameter changes of patient 1 (*middle top*) at a sample cross-sectional plane, namely the maximum diameter slice (P2). In addition, the spatial mean pressure variations over a cardiac cycle were calculated for the true lumen (*TL*) and false lumen (*FL*) separately, after which the pressure differences between 2 lumens were evaluated for each scan (*middle bottom*). Aortic growth rate (mm/year) at each follow-up scan, together with the maximum luminal pressure difference over a cardiac cycle were then evaluated and compared (*right*). The same procedure was repeated for all the other cross-sectional planes.

The first patient was a 39-year-old male who had a Bentall procedure with mechanical aortic valve replacement for TAAD. He underwent 5 follow-up CT scans over 3 years, the first of which was performed 4 months after initial operation. As shown in Figure 3, the maximum aortic diameter at a sample slice (P2) increased from 39.3 mm to 47.1 mm during the 3 years follow-up period. A faster aortic growth rate (3.5 mm/year) was observed earlier in the follow up that slowed down later (1.6 mm/year). Corresponding to the reduced growth rate, the maximum PD_TL-FL_ at P2 decreased from 12.4 to 4.6 mm Hg.

### Patient 2

**Figure 4:**
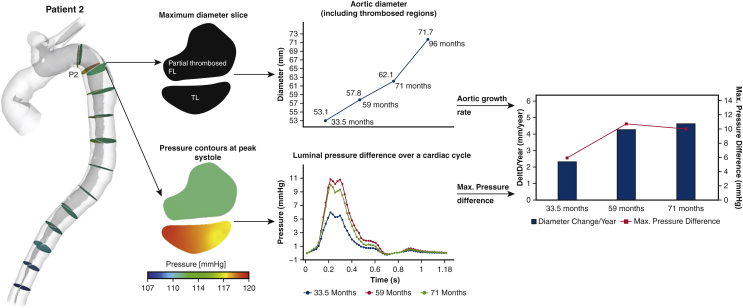
Illustration of aortic diameter changes of patient 2 (*middle top*) at the maximum diameter slice, together with true lumen (*TL*) and false lumen (*FL*) pressure difference variations over a cardiac cycle (*middle bottom*). Aortic growth rate (mm/year) at each follow-up scan, together with the maximum luminal pressure difference over a cardiac cycle were then evaluated and compared (*right*).

The second patient was a 48-year-old female with Marfan syndrome and hypertension who underwent replacement of the aortic root and ascending aorta for TAAD 33.5 months before initial follow-up scan. In contrast to patient 1, an initial slower aortic growth rate (2.3 mm/year) was identified, followed by a rapid aortic growth during late follow-ups, reaching 4.6 mm/year. A similar trend of increased PD_TL-FL_ was observed at the same cut plane (Figure 4).

### Patient 3

**Figure 5:**
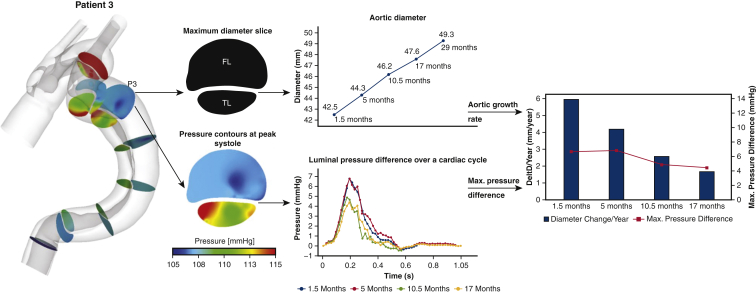
Illustration of aortic diameter changes of patient 3 (*middle top*) at the maximum diameter slice, together with true lumen (*TL*) and false lumen (*FL*) pressure difference variations over a cardiac cycle (*middle bottom*). Aortic growth rate (mm/year) at each follow-up scan, together with the maximum luminal pressure difference over a cardiac cycle were then evaluated and compared (*right*).

The third patient was a 72-year-old female with bicuspid aortic valve who was initially treated by replacing the aortic valve and ascending aorta for aortic valve stenosis and TAAD. Following the initial operation, 5 CTA examinations were performed for this patient over 27.5 months. The time interval between the surgery and first scan was 1.5 months. Similar to patient 1, the maximum aortic diameter (P3) increased from 42.5 mm to 49.3 mm, but the growth rate gradually slowed down from 6.1 mm/year to 1.7 mm/year between each 2 consecutive follow-up scans. Again, the maximum PD_TL-FL_ dropped from 6.75 to 4.42 mm Hg (Figure 5).

### Patient 4

**Figure 6:**
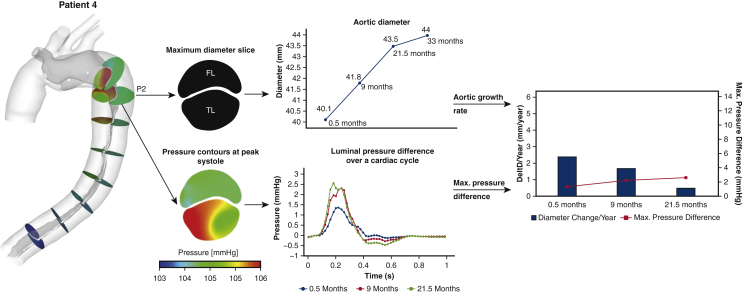
Illustration of aortic diameter changes of patient 4 (*middle top*) at the maximum diameter slice, together with true lumen (*TL*) and false lumen (*FL*) pressure difference variations over a cardiac cycle (*middle bottom*). Aortic growth rate (mm/year) at each follow-up scan, together with the maximum luminal pressure difference over a cardiac cycle were then evaluated and compared (*right*).

The fourth patient was a 79-year-old male who had emergency replacement of the aortic root and ascending aorta for TAAD. The first follow-up scan was taken at 0.5 month after surgery, after which 3 additional CTA examinations were performed over 33 months. He had a stable aortic growth from 40.1 mm to 44 mm. Although the maximum PD_TL-FL_ at the sample plane (P2) slightly increased from 1.37 to 2.58 mm Hg, these values were considerably steady compared to those in the other 3 patients. This patient had a steadily slow aortic growth rate (Figure 6).

### Linear Mixed Effects Analysis

**Figure E6:**
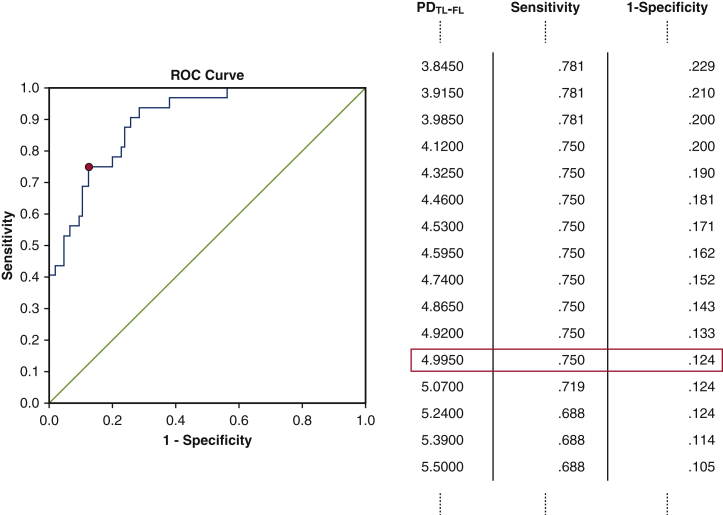
*Left*: illustration of fitted ROC curve with the optimal trade-off point being identified (as marked by *red dot*). *Right*: the coordinate of this point was then used to find the threshold value of PD_TL-FL_. *ROC*, Receiver operating characteristic; *PD*_*TL-FL*_, pressure difference between the true and false lumen.

The equivalent yearly aortic growth rate at each cross-sectional plane was evaluated and summarized in Table E2 (Appendix 3), together with the corresponding maximum PD_TL-FL_. We found that PD_TL-FL_ significantly predicted the aortic growth (coefficient, 0.26; 95% confidence interval, 0.15-0.37; *P* < .001). A table of solution for all fixed effects (Table E3) can be found in Appendix 4. Moreover, result from the fitted ROC curve (Figure E6, Appendix 4) shows that a maximum pressure difference higher than 5 mm Hg might cause unstable aortic growth.

## Discussion

The primary aim of surgery for TAAD is to identify and resect the primary entry tear, which is typically located at 2 to 5 cm above the sinotubular junction at the outer curvature of the ascending aorta.^17^ However, operative survival does not guarantee freedom from subsequent adverse aortic events, as 43% to 77.5% operative survivors have a patent FL.^2^, ^3^, ^4^ With a patent FL, aortic dilatation occurs in 49% to 100% patients, with a yearly mean growth rate of 1 to 5.6 mm.^2^, ^3^, ^4^ Timely reintervention could minimize the risk of sudden aortic rupture and late death. However, there are currently no reliable anatomical predictors of aortic size growth, and thus we chose to examine the hemodynamic impact on aortic dilatation based on patient-specific anatomical information and longitudinal CFD analysis. The aim of this study was to propose a CFD analysis technique that could predict future changes of the aortic size using standard clinical imaging techniques.

Pressure difference between TL and FL (PD_TL-FL_) is an important parameter driving movement of the intimal flap. Our previous study showed that the magnitude of PD_TL-FL_ was greater in patients with unstable aortic growth (mean yearly growth >2.9 mm) following TAAD surgical repair.^13^ The present study not only validates the previous findings but also shows the value of the technique in identifying previously stable patients at becoming high risk. Longitudinal analysis was therefore necessary to quantify the patient-specific values.

The results from the current study showed that greater PD_TL-FL_ is strongly associated with aortic dilatation because the aorta expanded faster at sites or time periods of higher luminal pressure difference. As shown in Figure 3, Figure 4, Figure 5, Figure 6, aortic growth rate of each patient varies at different time periods and variations of PD_TL-FL_ follow the same pattern. Furthermore, comparing the aortic growth rate among various aortic segments (Appendix 3), aortic expansion was greater in the proximal region (eg, P1-P4) than distal region. This could also be linked with decreased luminal pressure difference from the proximal portion to distal portion. ROC curve shows that unstable aortic growth (>2.9 mm/year) might be driven by a PD_TL-FL_ value greater than 5 mmHg. However, this finding needs to be further validated with large patient cohorts. Linear mixed effects analysis revealed that PD_TL-FL_ was a statistically significant predictor of aortic growth (*P* < .001).

Pressure difference between the true and false lumen is affected by the number and size of tears. The lack of distal tears in TBAD was reported to increase FL pressure while the presence of at least one large tear or multiple re-entry tears along the length of dissection could equalize the pressure between 2 lumens.^18^^,^^19^ In our study, the number and size of tears were measured (Table 1) to examine their impacts on luminal pressure difference. Patient 1 had a remaining entry tear located distally to the left subclavian artery and the number of distal tears increased over time. This helped alleviate the pressure in FL, thereby reducing luminal pressure difference. Although the FL was partially thrombosed, it was confined to the distal descending thoracic aorta (eg, P4-P8) and did not occlude the distal tears. This might explain why it did not cause any late adverse outcomes as reported by Tsai and colleagues.^20^ In their TBAD study, partial thrombosis impeding outflow was associated with significantly elevated diastolic pressure in FL, which accounts for FL dilatation and aneurysm formation.^21^ In contrast to their studies, we found that aortic dilatation was mainly correlated to a greater PD_TL-FL_ during systole. This is reasonable since hemodynamics in repaired TAAD are significantly different from TBAD.^22^

Numerous re-entry tears were observed on the initial follow-up scan of patient 2, and a few of them disappeared on subsequent follow-up scans, which might have been occluded by thrombus formation in the FL. FL partial thrombosis mainly occurred in the proximal descending thoracic aorta (eg, P1-P5) of patient 2. Following the first scan, luminal pressure difference rose significantly in this region since all the tears were occluded and there was no flow exchange between the 2 lumens. In fact, an obvious drop in PD_TL-FL_ was observed in the distal region (eg, below P5) where flow exchange restored.

No partial thrombosis was observed for patients 3 and 4. Patient 3 presented with a larger number of re-entry tears on the initial scan compared with the second and third follow-ups but also showed larger PD_TL-FL_ and greater aortic growth. Nevertheless, results are also inconsistent in the literature since a greater number of re-entry tears has also been reported to cause greater growth rate.^23^ It should be mentioned that there is an exceptional cut plane (P1) with stable aortic growth but larger luminal pressure difference (Appendix 3). P1 located at an extremely curved aortic segment where aortic expansion was constrained. Patient 4 also had a larger number of re-entry tears along the dissected aorta on the first scan, which resulted in small luminal pressure difference. However, by comparing the TL/FL volume changes between the first and second scans, there was a significant compression of the TL and FL expansion, which is an adverse prognostic sign. Considering the first scan was taken only 15 days after surgery, surgery-induced wall remodeling might still be in progress. In fact, a reverse trend of compression of the FL and TL expansion was found during subsequent scans.

### Clinical Relevance

To our knowledge, the results of this study are the first to systematically demonstrate the relationship between PD_TL-FL_ and aortic dilatation using clinical image data of repaired TAAD patients over time in a follow-up period. Our published results^13^ revealed a strong association between aortic dilatation and luminal pressure difference. The current study not only shows that PD_TL-FL_ was a statistically significant predictor of aortic growth (linear mixed effects analysis, *P* < .001) but also identifies a potential threshold value of 5 mm Hg for PD_TL-FL_ to predict which patient is likely to experience unstable aortic growth. However, this threshold value was obtained based on limited sample size and thus needs to be validated in future studies by involving large patient cohort. With the methodology proposed in our present and previous studies,^13^ a prospective study should be designed to further validate the value of the technique in predicting response to various surgical strategies.

### Limitations

There are several limitations to the present study. First, we only included 4 patients with heterogenous clinical backgrounds, including differences in aortic anatomy and operative approach and also a history of Marfan syndrome in 1 patient. However, the serial imaging analysis of each patient based on multiple follow CTA scans allowed a longitudinal comparison, and thus weakening the compound effects of various patients’ background.

The CFD technique is currently time-consuming, thereby limiting the number of cases included in this study (4 patients, each with 4-5 follow-up scans, providing a total of 18 simulated cases). The computational time may be reduced by developing artificial intelligence techniques to automate the workflow and computational procedures, and luminal pressure estimation to allow the application of the technique in routine clinical practice in the future. The knowledge gained through this study will also help to develop new strategies aimed at minimizing luminal pressure difference, thereby reducing the risk of subsequent complications.

In terms of the computational model, the aortic wall was assumed to be rigid. In reality, the aorta expands and contracts in response to the pulsation of blood pressure, rendering the use of fluid–structure interaction to account for the dynamic effects of moving wall on blood flow.^24^ However, performing a fluid–structure interaction simulation is computationally very demanding due to the complexity of the computation, and thus not feasible for multiple case studies and particularly in routine clinical practice. Finally, for each outlet, a 3-element Windkessel model was applied to account for the behavior of the distal vascular bed and to predict the physiologically realistic pressure waveforms. However, patient-specific pressure data were not available for estimation of 3-element Windkessel model parameters, which may influence the patient specificity of the predicted pressure values but should not affect the evaluated pressure difference.^25^

## Conclusions

Assessment of pressure differences by CFD in the true and false lumens following TAAD repair appears to predict the risk of aortic dilatation over time. This is a promising biomarker to allow identification of patients at high risk of progressive aortic dilatation following surgical ascending aorta replacement. In future studies, we will examine if CFD can predict the outcome of surgical repair, based on preoperative treatment simulations and resultant pressure changes.

### Conflict of Interest Statement

The authors reported no conflicts of interest.

The *Journal* policy requires editors and reviewers to disclose conflicts of interest and to decline handling or reviewing manuscripts for which they may have a conflict of interest. The editors and reviewers of this article have no conflicts of interest.

## Figures and Tables

**Table E1:** Comparison of key geometric parameters between the original and repeated reconstructions

	Patient 3					
	Scan 2			Scan 3		
	Original model	Repeated model	Difference (%)	Original model	Repeated model	Difference (%)
Geometric parameters						
TL volume (including the ascending aorta, mm^3^)	109097	105423	3.4	108226	107100	1.0
FL volume, mm^3^	167692	163288	2.6	169649	166752	1.7
Total volume, mm^3^	276789	268711	2.9	277875	273852	1.4
Primary tear size, mm^2^	69	71	2.9	71	70	1.4
Distance between primary tear and LSCA, mm	63.3	63.8	0.8	61.1	58.9	3.6
Aortic diameters, mm						
P1	27.8	27.4	1.4	27.7	27.9	0.7
P2	40.9	40.6	0.7	43.6	43.4	0.5
P3	44.3	44.1	0.5	46.2	46.0	0.4
P4	37.2	36.6	1.6	38.6	38.1	1.3
P5	33.2	32.8	1.2	33.2	32.7	1.5
P6	30.2	29.9	1.0	29.6	29.4	0.7
P7	27.9	27.5	1.4	27.9	27.7	0.7
P8	27.6	27.3	1.1	27.4	27.1	1.1

Monthly aortic growth rate, mm/mo	Original model	Repeated model	Difference (%)
P1	0	0.09	–
P2	0.49	0.51	4.1
P3	0.32	0.35	9.4
P4	0.25	0.27	8
P5	0	0	–
P6	0	0	–
P7	0	0.04	–
P8	0	0	–

*TL*, True lumen; *FL*, false lumen; *LSCA*, left subclavian artery.

**Table E2:** Monthly and equivalent yearly aortic growth rate together with the corresponding maximum luminal pressure difference at each cross-sectional plane

Patient 1												
	P1	P2	P3	P4	P5	P6	P7	P8	P9	P10	P11	P12
20161027	33.8	39.3	36.1	35.3	35.9	34.5	30.7	30.6	30	20.2	20.7	22.6
20170508	40.1	41.2	38.3	38.9	37.3	33.1	31	30.5	31.8	21.6	22.9	24.6
Δ*D*/month, mm	0.97	0.29	0.34	0.55	0.22	0	0.05	0	0.28	0.27	0.34	0.21
Equivalent Δ*D*/year, mm	11.6	3.5	4.1	6.6	2.6	0	0.6	0	3.4	3.2	4.1	2.5
Maximum (TL-FL) pressure, mm Hg	10.3	12.41	11.98	10.22	7.55	7.68	5.84	5.41	5.59	7.18	2.38	0.23
20170508	40.1	41.2	38.3	38.9	37.3	33.1	31	30.5	31.8	21.6	22.9	24.6
20171114	42.3	43.6	40.1	40.2	40.3	35.9	32.7	33.1	33.2	22	24.7	26.4
Δ*D*/month, mm	0.37	0.4	0.3	0.28	0.5	0.47	0.28	0.43	0.23	0.07	0.3	0.22
Equivalent Δ*D*/year, mm	4.4	4.8	3.6	3.4	6	5.6	3.4	5.2	2.8	0.8	3.6	2.6
Maximum (TL-FL) pressure, mm Hg	9.31	9.85	9.64	7.81	5.65	5.76	4.01	3.67	3.87	6.47	1.43	0.09
20171114	42.3	43.6	40.1	40.2	40.3	35.9	32.7	33.1	33.2	22	24.7	26.4
20181106	43.7	45.2	41	42	41.4	38.4	30.8	34.2	33.6	22.9	26	27.9
Δ*D*/month, mm	0.12	0.13	0.08	0.15	0.09	0.21	0	0.09	0.03	0.08	0.11	0.13
Equivalent Δ*D*/year, mm	1.4	1.6	1.0	1.8	1.1	2.5	0	1.1	0.4	1	1.3	1.6
Maximum (TL-FL) pressure, mm Hg	5.37	6.05	4.96	3.87	2.71	2.47	1.16	1.4	1.01	3.02	0.59	0.07
20181106	43.7	45.2	41	42	41.4	38.4	30.8	34.2	33.6	22.9	26	27.9
20191106	41.9	47.1	42.3	42.3	42.4	40	26.4	35.6	34.4	23.7	26.4	29.4
Δ*D*/month, mm	0	0.16	0.11	0.03	0.08	0.13	0	0.12	0.07	0.07	0.03	0.13
Equivalent Δ*D*/year, mm	0	1.9	1.3	0.4	1.0	1.6	0	1.4	0.8	0.8	0.4	1.6
Maximum (TL-FL) pressure, mm Hg	3.96	4.56	3.49	2.47	2.21	2.21	1.1	1.55	1.1	2.53	0.25	0.05

Patients 2												
	P1	P2	P3	P4	P5	P6	P7	P8	P9	P10	P11	P12
20150629	43.5	53.1	44.6	30.3	31.5	32.2	30.6	29.2	25	26.9	26.8	24
20170808	46.7	57.8	51	32.4	33.4	33.9	30.8	30	27.4	28.9	28.3	23.9
Δ*D*/month, mm	0.13	0.19	0.26	0.08	0.07	0.07	0.01	0.03	0.1	0.08	0.06	0
Equivalent Δ*D*/year, mm	1.6	2.3	3.1	1.0	0.8	0.8	0.1	0.4	1.2	1.0	0.7	0
Maximum (TL-FL) pressure, mm Hg	6.46	5.94	5.03	4.5	4.23	1.59	-1.7	-0.7	0.65	0.5	0.12	0.17
20170808	46.7	57.8	51	32.4	33.4	33.9	30.8	30	27.4	28.9	28.3	23.9
20180809	50.9	62.1	54.7	34.6	34.7	35.1	32.7	30.6	27.9	28.3	28.8	23.1
Δ*D*/month, mm	0.35	0.36	0.3	0.19	0.11	0.1	0.16	0.05	0.04	0	0.04	0
Equivalent Δ*D*/year, mm	4.2	4.3	3.6	2.3	1.3	1.2	1.9	0.6	0.5	0	0.5	0
Maximum (TL-FL) pressure, mm Hg	10.83	10.74	9.74	8.64	7.92	4.63	-4.2	-1.7	0.67	0.31	-0.3	0.2
20180809	50.9	62.1	54.7	34.6	34.7	35.1	32.7	30.6	27.9	28.3	28.8	23.1
20200914	65.9	71.7	62.1	36.1	37.1	36.5	33.5	32.8	29.7	N/A	N/A	N/A
Δ*D*/month, mm	0.6	0.38	0.3	0.06	0.1	0.06	0.03	0.09	0.07	N/A	N/A	N/A
Equivalent Δ*D*/year, mm	7.2	4.6	3.6	0.7	1.2	0.7	0.4	1.1	0.8	N/A	N/A	N/A
Maximum (TL-FL) pressure, mm Hg	10.5	10.05	8.71	7.52	6.35	3.17	-2.3	1.1	0.71	N/A	N/A	N/A

Patient 3								
	P1	P2	P3	P4	P5	P6	P7	P8
20170202	27	36.4	41	35.8	31.8	29.2	27.1	26.1
20170518	27.8	40.9	44.3	37.2	33.2	30.2	27.9	27.6
Δ*D*/month, mm	0.23	0.71	0.51	0.4	0.4	0.21	0.23	0.38
Equivalent Δ*D*/year, mm	2.8	8.5	6.1	4.8	4.8	2.5	2.8	4.6
Maximum (TL-FL) pressure, mm Hg	−2.44	5.87	6.75	3.82	3.44	2.42	1.79	2.82
20170518	27.8	40.9	44.3	37.2	33.2	30.2	27.9	27.6
20171101	27.7	43.6	46.2	38.6	33.2	29.6	27.9	27.4
Δ*D*/month, mm	0	0.49	0.35	0.25	0	0	0	0
Equivalent Δ*D*/year, mm	0	5.9	4.2	3	0	0	0	0
Maximum (TL-FL) pressure, mm Hg	–4.78	6.37	6.86	3.69	3.63	2.36	1.7	3.73
20171101	27.7	43.6	46.2	38.6	33.2	29.6	27.9	27.4
20180518	28.2	46.2	47.6	39.4	33.2	29.6	27.8	27.7
Δ*D*/month, mm	0.08	0.4	0.22	0.12	0	0	0	0.05
Equivalent Δ*D*/year, mm	1.0	4.8	2.6	1.4	0	0	0	0.6
Maximum (TL-FL) pressure, mm Hg	–7.33	5.11	4.88	2.61	1.28	2.35	–1.96	3.1
20180518	28.2	46.2	47.6	39.4	33.2	29.6	27.8	27.7
20190517	27.8	48.5	49.3	41.1	33.1	29.8	28.5	28.5
Δ*D*/month, mm	0	0.2	0.14	0.14	0	0.02	0.06	0.07
Equivalent Δ*D*/year, mm	0	2.4	1.7	1.7	0	0.2	0.7	0.8
Maximum (TL-FL) pressure, mm Hg	-5.14	4.85	4.42	2.16	2.12	1.06	0.5	2.51

Patient 4								
	P1	P2	P3	P4	P5	P6	P7	P8
20150416	31.3	40.0	37.1	32.7	32.4	30.0	29.5	29.8
20160104	33.0	41.8	39.0	33.3	32.9	30.4	29.8	29.8
Δ*D*/month, mm	0.2	0.2	0.22	0.07	0.06	0.05	0.04	0
Equivalent Δ*D*/year, mm	2.4	2.4	2.6	0.8	0.7	0.6	0.5	0
Maximum (TL-FL) pressure, mm Hg	1.47	1.37	1.46	0.42	0.69	0.2	0.12	0.22
20160104	33.0	41.8	39.0	33.3	32.9	30.4	29.8	29.8
20170120	33.8	43.5	40.0	34.2	33.4	30.6	29.8	29.9
Δ*D*/month, mm	0.07	0.14	0.08	0.08	0.04	0.01	0	0.01
Equivalent Δ*D*/year, mm	0.8	1.7	1.0	1.0	0.5	0.1	0	0.1
Maximum (TL-FL) pressure, mm Hg	2.24	2.25	2.63	2.28	1.62	0.79	0.55	0.32
20170120	33.8	43.5	40.0	34.2	33.4	30.6	29.8	29.9
20180108	34.0	44.0	40.6	34.4	32.7	32.3	30.5	30.1
Δ*D*/month, mm	0.02	0.04	0.05	0.02	0	0	0	0
Equivalent Δ*D*/year, mm	0.2	0.5	0.6	0.2	0	0	0	0
Maximum (TL-FL) pressure, mm Hg	2.33	2.58	2.79	2.25	1.71	1.07	0.63	0.34

**Table E3:** Solution for fixed effects. The results were obtained using SAS, v. 9.4 (SAS Institute Inc)

## Appendix 2. Intraoperator Reproducibility Study

An intraoperator reproducibility study was carried out to analyze how repeatable the geometry reconstructions in this study were. Two geometries from computed tomography angiography scans of patient 3 (scans 2 and 3) were reconstructed again by the same operator, without referencing the original reconstructions used in the computational fluid dynamics simulations. Key geometric parameters were measured and compared between the original and repeated reconstructions. The results are summarized in Table E1.

## Appendix 4. Statistical Analysis

### Three-level Linear Mixed Model

#### Data structure and equations

The corresponding 3-level equations are listed to follow:

(1) $$Level\;1:Y_{ijk}=b_{jk}+\beta_{jk}+e_{ijk}$$

(2) $$Level\;2:b_{jk}=b_{k}+\mu_{j\left(k\right)}$$

(3) $$\beta_{jk}=\beta_{k}+\beta_{3j\left(k\right)}$$

(4) $$Level\;3:b_{k}=\beta_{0}+v_{k}$$

(5) $$\beta_{k}=\beta_{3}+\beta_{3k}$$

Where *Y*_*ijk*_ is the aortic growth rate at scan time *i* at the *j*^*th*^ location of the *k*^*th*^ patient, and *v*_*k*_, *μ*_*j*(*k*)_, *e*_*ijk*_ are residuals. By substituting equations (2), (3), (4), (5) into equation 1, the final equation can be written as:

$$Y_{ijk}=\beta_{0}+\beta_{1}*time+\beta_{2}*location+\left(\beta_{3}+\beta_{3k}+\beta_{3j\left(k\right)}\right)*PD_{TL-FL}+v_{k}+\mu_{j\left(k\right)}+e_{ijk}$$

### Receiver Operating Characteristic (ROC) Curve Fitting

A ROC curve (Figure E6) was fitted in SPSS, v. 23.0 (IBM Corp) to find a threshold value of pressure difference between the true and false lumen (PD_TL-FL_) that may predict which patient is likely to present unstable aortic growth (>2.9 mm/year). The red dot in Figure E6 (left) represents the optimal trade-off point, which should have the highest possible values for sensitivity and specificity simultaneously. The coordinate of this point was then used to find the threshold value of PD_TL-FL_.

## Supplementary Data

Video 1 Background, methods (patient-specific modelling), key findings, and the potential clinical implications of this study are summarized. Video available at: https://www.jtcvs.org/article/S2666-2736(22)00035-3/fulltext.
