# Supplementary figures and images for: Prediction of aortic dilatation in surgically repaired type A dissection: A longitudinal study using computational fluid dynamics
Source: JTCVS Open. 2022 Feb 9;9:11–27. doi: 10.1016/j.xjon.2022.01.019 (PMC9390758; doi:10.1016/j.xjon.2022.01.019)

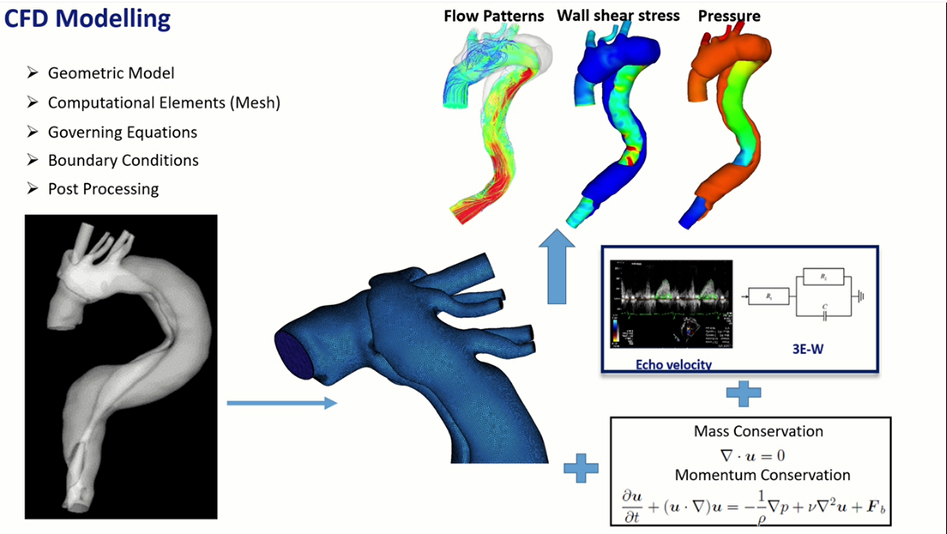

Supplement: Video 1 — Background, methods (patient-specific modelling), key findings, and the potential clinical implications of this study are summarized. Video available at: https://www.jtcvs.org/article/S2666-2736(22)00035-3/fulltext. [file fx3.jpg]
